# Coffee by-products as the source of antioxidants: a systematic review

**DOI:** 10.12688/f1000research.107811.1

**Published:** 2022-02-23

**Authors:** Wahyu Lestari, Kartini Hasballah, M. Yulianto Listiawan, Sofia Sofia

**Affiliations:** 1 Postgraduate Program, School of Medicine, Universitas Syiah Kuala, Banda Aceh, 23111, Indonesia; 2 Department of Dermatology, School of Medicine, Universitas Syiah Kuala, Banda Aceh, 23111, Indonesia; 3 Department of Dermatology, Dr. Zainoel Abidin General Hospital, Banda Aceh, 24415, Indonesia; 4 Department of Pharmacology, School of Medicine, Universitas Syiah Kuala, Banda Aceh, 23111, Indonesia; 5 Department of Dermatology, Faculty of Medicine, Universitas Airlangga/Dr. Soetomo General Hospital, Surabaya, 60131, Indonesia; 6 Department of Biochemistry, School of Medicine, Universitas Syiah Kuala, Banda Aceh, 23111, Indonesia; 7 Master of Public Health, School of Medicine, Universitas Syiah Kuala, Banda Aceh, 23111, Indonesia

**Keywords:** robusta, arabica, husk, pulp, silverskin, cascara

## Abstract

**Background:** Solid waste from coffee depulping process threatens the organism in environment as it produces organic pollutants. Evidence suggested that coffee by-product could valorize owing to its potential as antioxidant sources. The aim of this systematic review was to evaluate antioxidant activity of coffee by-products obtained from different coffee variants (arabica and robusta) and processing methods.

**Methods:** The systematic review was conducted as of May 29, 2021 for records published within the last ten years (2011–2021) using seven databases: Embase, Medline, BMJ, Web of Science, Science Direct, Cochrane, and PubMed. Data on type of specimen, processing methods, and antioxidant activities were collected based on PRISMA guidelines.

**Results:** Our data suggested that aqueous extract was found to be the most common processing method used to obtain the antioxidant from various coffee by-products, followed by methanol and ethanol extract. A variety of antioxidant properties ranging from strong to low activity was found depending on the variety, type of coffee by-products (cascara, pulp, husk, silverskin, and parchment), and processing technique. Fermentation employing proper bacteria was found effective in improving the yield of bioactive compounds resulting in higher antioxidant capacity. Applications in feedstuffs, foods, beverages, and topical formulation are among the potential utilization of coffee by-products.

**Conclusion:** Coffee by-products contain bioactive compounds possessing antioxidant properties which could be used as additives in foods, beverages, and cosmetics. In particular, their benefits in skin care products require further investigation.

## Introduction

As the world widely popular beverage, ^1^^,^^2^ coffee has been produced in a large scale causing the emergence of massive organic solid waste. ^3^^,^^4^ Most of the solid waste is originated from the depulping process, where the coffee bean is separated from the other parts of the coffee cherry. ^5^^,^^6^ Solid waste from cherry pulp was not well-managed leading to the threat of environmental pollution. ^7^ Nevertheless, the solid waste can be utilized for multiple purposes such as bioethanol, ^8^ biogas, ^9^ compost, ^10^ and feedstuffs. ^11^^,^^12^ Coffee by-products consist of cascara, coffee pulp, coffee husk, coffee silverskin, and coffee parchment. Among them, coffee pulp occupies the most part of coffee by-products containing carbohydrate (50%), protein (10%), fiber (20%), fat (2.5%), caffeine (1.3%), and phenolic compounds. ^13^ The phenolic acids in cherry pulp can be detailed as followed; hydro-benzoic acid, chlorogenic acid, ferulic acid, caffeic acid, syringic acid, gallic acid, vanillic acid, and cumaric acid. ^12^^,^^14^ Many studies have been conducted in coffee. ^15^^–^^20^

In dry processing, the solid waste majorly produced is husk. ^6^ Coffee husk is rich in carbohydrate (8–85%), followed by protein (8–11%), fat (0.5–3%), and minerals (3–7%). ^21^ Cascara obtained from husk or pulp contains natural antioxidants namely polyphenols, anthocyanin, and vitamin C along with other bioactive compounds of caffeine, alkaloids, and tannins. ^22^ Husk is potential for human consumption due to its nature of free gluten which does not cause allergic reaction. ^21^

Taken together, coffee by-products hold a significant potential to be utilized as additives in food products. ^23^^,^^25^ In fact, foods and beverages derived from coffee by-product have been introduced and recorded in scientific report a long time ago. ^12^ In dermatology, the antioxidant properties from coffee by-products could provide skin protection against UV light-induced damages. ^26^^,^^27^ Moreover, the content of polyphenols could be used for patient with alopecia, acne vulgaris, fungal infection, hyperpigmentation, or skin aging. ^28^ Valorization of coffee by-products in a wide array of fields could offer a solution to the emerging environmental threat due to the overwhelming production of coffee solid waste. ^24^^,^^29^

So far, the review of coffee by-products only presents the end products in general. ^10^^,^^24^^,^^30^ Most of the reviews discussed about the application of coffee by-products in food and beverage products. ^12^^,^^31^ Review of coffee-by products with specific topics such as topical formulation ^32^ and polymer technology ^5^ have been reported. Herein, we discuss the advances of coffee by-products antioxidant activities which were obtained from different coffee variants (arabica and robusta) and processing methods. To the best of our knowledge, the systematic review of antioxidant activities yielded by coffee by-products has never been published.

## Methods

### Study setting and eligibility criteria of studies

This systematic review was conducted in accordance to the Preferred Reporting Items for Systematic Reviews and Meta-analyses (PRISMA) guidelines ^33^ as previously used elsewhere. ^34^^,^^35^ Articles were included in this review, when the following criteria were fulfilled: 1). The sample was at least coffee husk, coffee silverskin, coffee pulp, coffee parchment, or the cascara (pulp and outer skin); 2). Investigated *in-vitro* or *in-vivo* anti-oxidant activities using standardized methods, as reported previously ^36^; and 3). Published in the last 10 years (2011–2021) and written in English or Indonesian Language. Studies that only determined total phenolic compounds were not included. Editorials, reviews, commentaries, case reports, book or book chapter were excluded.

### Database and search strategy

The search was conducted in May 29, 2021 through search engines of the following databases: Embase, Medline, BMJ, Web of Science, Science Direct, Cochrane, and PubMed. The terms combination used to search in the title, abstract, and keywords was “((cascara coffee) OR (coffee husk) OR (coffee pulp) OR (coffee waste)) AND ((antioxidant) OR (photoaging))”.

### Study selection and data extraction

A reference manager (EndNote X9, Thompson Reuters, Philadelphia, PA, USA) was used to import the list of references from all databases, where duplicates were then removed. The two-steps selection was carried out by firstly remove the non-eligible article by screening the titles and abstract from the collected references. Secondly, two authors WL and SS conducted the screening of the full texts according to the stated inclusion criteria and data availability. The data were extracted from main articles and their supplementary materials, whenever required. The extracted data included the report characteristics (author/s, publication year), type of specimen (coffee husk, coffee silverskin, coffee pulp, coffee parchment or cascara), processing methods (pre-treatment and extraction), and outcomes (antioxidant activity and others).

## Results

### Study eligibility results

**Figure 1.**
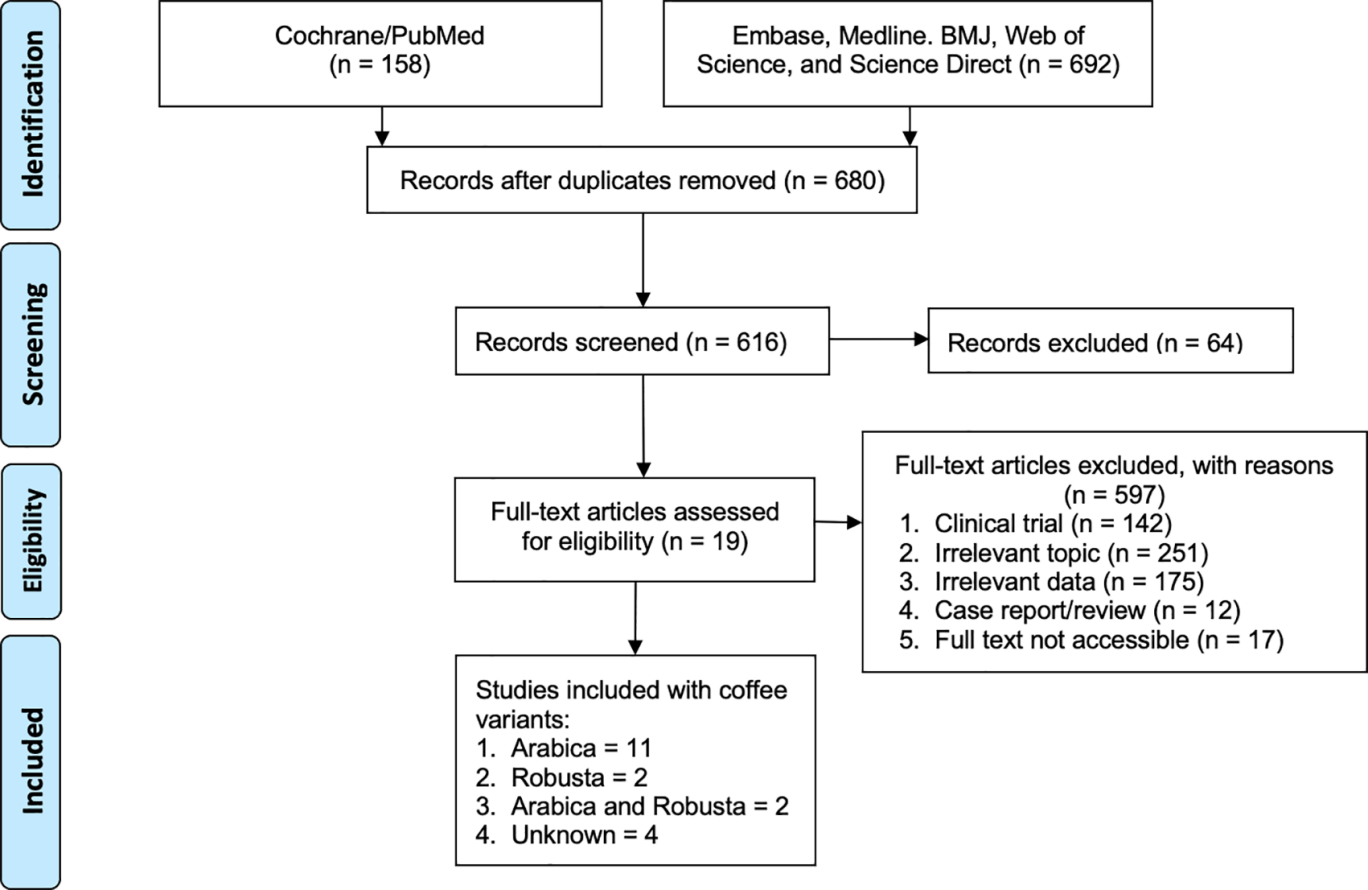
Schematic diagram of the literature search according to PRISMA.

The search yielded 850 records from the stated databases ( Figure 1), where as many as 170 duplicates were removed. The duplicate removal left 680 articles to undergo the first screening, of which, 616 studies were potentially eligible. Second screening excluded 597 articles, resulting the final 19 articles for qualitative synthesis.

Most of the studies produced crude extract using water, ^22^^,^^37^^,^^44^ followed by extraction using polar extracts (such as methanol and ethanol) ^38^^,^^40^^,^^45^^,^^46^ and non-polar extract (n-hexane). ^47^ A study, in particular, used supercritical fluid extraction with CO _2_ as the solvent. ^48^

### Antioxidant properties of coffee by-products

The investigations of antioxidant activities were varied in each study, but mostly, DPPH assay was employed ( Table 1). ^6^^,^^22^^,^^40^^,^^44^^–^^46^^,^^48^^,^^52^ Based on DPPH assay, the IC _50_ reached as low as 5.8 ppm obtained in the lotion product made of coffee pulp extract using ethanol and water solvent. ^46^ However, the value is hard to compare since other studies did not report the IC _50_ and used different methods. Other antioxidant assays included FRAP, ^37^^,^^38^^,^^42^^,^^43^ ABTS, ^38^^,^^43^^,^^45^^,^^49^^,^^53^ NO, ^45^^,^^47^ ORAC ^42^^,^^51^ and Folin-Ciocalteu. ^45^ Pre-treatments such as fermentation, ^47^^,^^50^ vacuum drying, ^52^ sun-drying, ^37^ and lipophilization ^42^^,^^49^ were found to yield the optimum value of antioxidant capacity. Other than crude extract, the reports also investigated the antioxidant properties of products made by coffee cascara such as dietary fiber, ^53^ instant beverage powder ^43^ lotion, ^46^ fodder, ^37^ anthocyanin, ^44^ and essential oil. ^6^^,^^51^

**Table 1.** Antioxidant properties of coffee by-products.

Year	By-product	Processing	Antioxidant properties *	Others	Reference
2018	Arabica coffee husk	Hydrodistillation extraction to obtain essential oil	84.60% at 100 ppm ^a^	The oil was dominated by aromatic compounds, in which 30% and 40% of the total compounds were hydrocarbon and oxygenated constituents, respectively.	^6^
2018	Arabica coffee pulp	Ensiling and sun-drying to obtain fodder	>2,5 μmol TE ^-1^ mL ^b^	Ensiled sun-dried coffee pulp has the highest crude protein, neutral detergent fiber, and acid detergent fiber	^37^
2019	Arabica and robusta coffee pulp	Extraction using water at different temperatures	33.5% at 100 ppm ^a^	Best phenolic content was obtained at 85°C Best antioxidant activities were obtained at 75°C Antibacterial activities of arabica are better than robusta	^22^
2020	Robusta coffee pulp	Hot air drying, vacuum drying and low temperature and pressure drying	Antioxidant capacity (mg TE/g DW): 21.39 ^c^, 2.24 ^a^, and 16.73 ^b^	Vacuum drying is the optimum method, resulting the highest contents of phenolics, caffeine, gallic acid, and proanthocyanins.	^52^
2020	Arabica coffee husk	Extraction using water, ethanol, and combination of water and ethanol	Antioxidant capacity at 100 ppm: 84.95% ^a^, 97.21% ^c^, and 3136.4 μmol TE/g ^b^	Water bath with water and ethanol (1:1) extraction yielded the highest bioactive compounds (phenolics, flavonoids, and tannins).	^38^
2017	Arabica coffee pulp	Extraction using water	51 – 92 μmol TE/g DM ^c^	The caffeine amounting up to 226 mg/L and total polyphenols – 283 mg GAE/L	^39^
2011	Arabica coffee husk and coffee ground	Supercritical fluid extraction (SFE) using CO _2_ and CO _2_ with co-solvent	IC _50_ > 250 μg/mL ^a^	The best method is low pressure extraction. Dominating compounds of the extract include caffeine and chlorogenic acid.	^48^
2015	Coffee husk	Extraction using combination of methanol, ethanol or water	IC _50_ < 25 μg/mL ^a^ IC _50_ < 30 μg/mL ^b^	Samples extracted using methanol (50%) has the highest phenolic contents and antioxidant activity.	^40^
2020	Arabica coffee pulp	Isolation of volatile and non-volatile compounds	35.8 μmol TE/g ^a^ 12.23 μmol TE/g ^e^	151 volatile compounds had been obtained (mainly alcohols, acids, ketones, and esters)	^51^
2020	Robusta coffee pulp	Extraction of free and bound phenolics using ethanol and combination of ethanol and ethyl acetate, respectively	IC _50_ = 12.75 μg/mL ^a^ IC _50_ = 30.76 μg/mL ^c^ 6.38 M TE/100g ^b^	The coffee pulp extract could be used as the source of pectin and polyphenols with good antioxidant activities	^45^
2018	Arabica coffee pulp	Extraction using combination of water and ethanol	44.49 mg GAE/g ^f^ 141.7 μmol TE/g ^a^	The coffee pulp extracts reduce the production of IL-8 in gastric epithelial cells.	^45^
2019	Arabica coffee pulp	Phenolic extraction using water or HCl 1%	57 087.8 μmol TE/100g ^b^ 806.93 mg AEAC/100g ^e^	Extraction using HCl yielded the best overall activities	^42^
2020	Arabica cascara	Freeze-dried aqueous extract of coffee cascara	82.85 mq eq. CGA/m L ^c^ 1.08 mg eq. CGA/mL ^b^	Melanoidins were correlated to the color of infused cascara beverage. The instant cascara beverage has low acrylamide and caffeine levels with many antioxidants and nutrients.	^43^
2012	Coffee pulp	Anthocyanin purification using column extraction	90% at 200 ppm ^a^	The anthocyanin could be retrieved from coffee pulp to produce food colorants and bioactive ingredients	^44^
2020	Coffee pulp	Fermentation of coffee pulp using indigenous lactic acid bacteria	42.6% at 100 ppm ^a^	Fermentation using indigenous lactic acid produced coffee pulp with higher antioxidant activity	^50^
2020	Coffee pulp	Extraction using ethanol: water solvent to produce lotion	IC _50_ = 5.8 ppm ^a^	The stability and antioxidant activity of the lotion containing coffee pulp extract were higher in comparison to the commercial product.	^46^
2016	Arabica coffee pulp	Drying and blending followed by aqueous extraction and lyophilization	IC _50_ = 82 μg/mL ^a^ IC _50_ = 18 μg/mL ^c^	Possessing antibacterial activity against gram-positive and negative bacteria.	^49^
2010	Arabica and robusta coffee pulp, husk, parchment husk, silverskin, and coffee ground	Isolation of dietary fiber	2.12 mmol TE/100g ^c^	Dietary fiber retrieved from coffee by-products is rich with bioactive compounds and high antioxidant activity.	^53^
2011	Arabica coffee pulp	Fermentation using *Aspergillus tamarii,* followed by hexane extraction	ED _50_ = 0.034 mg ^d^ t _ED50_ = 1.12 minutes ^d^	Higher antioxidant activity in the fermented coffee pulp	^47^

GAE: gallic acid equivalent; TE: Trolox equivalent.

* The most optimal value from the obtained product.

Determined by

^a^ 2,2-diphenyl-1-picrylhydrazyl (DPPH) assay;

^b^ ferric reducing antioxidant power (FRAP) assay;

^c^ 2,2′-azino-bis (3-ethylbenzothiazoline-6-sulfonic acid) (ABTS) assay;

^d^ nitric oxide (NO) assay;

^e^ oxygen radical absorbance capacity (ORAC) assay;

^f^ Folin-Ciocalteu assay.

## Discussion

Treated as a solid waste in coffee industry, coffee by product could be a threat to the environment, especially to the aquatic organisms. ^7^ Nonetheless, they could be used as the source of antioxidant into various food and beverage products, *viz* tea, ^43^^,^^54^^,^^55^ dietary fiber, ^56^^,^^57^ food preservatives, ^58^^,^^59^ wheat flour substitute, ^60^ and food additives. ^61^^,^^62^ Consumption of foods or beverages derived from coffee by-products has been associated to its health benefits owing to the rich presence of bioactive compounds. Phenolic compounds and caffein have been reported contained in the coffee pulp of arabica and robusta variants. ^63^ Phenolic compounds in high concentrations might also be retrieved from arabica coffee silverskin. ^64^

A study comparing robusta and arabica coffee pulp with aqueous extraction revealed higher antioxidant capacity in that of arabica variant. ^22^ However, a different result was shown by another study comparing the antioxidant activities of coffee silverskin from both variants. The study found that coffee silverskin from robusta variant has higher antioxidant activity as suggested by DPPH, ABTS, and FRAP assays. ^65^ Higher antioxidant efficacies of robusta variant were also revealed by a study employing green coffee extract. ^66^ Antioxidant activities could be affected not only by the variant, but also the extraction or brewing method. ^67^ Additionally, each coffee by-product could have different levels of antioxidant activity, where coffee silverskin was revealed to have the highest value. ^53^

Water has been a common solvent used on coffee by products, as reported by many researches. ^22^^,^^39^^,^^43^^,^^52^ This due to the fact that aqueous extraction is the most practical processing method of obtaining antioxidant compounds. Moreover, it may also attract polar and semi-polar compounds such as phenolic acids, flavonoids, and so on. ^68^^,^^69^ Other studies combined water with methanol or ethanol which can increase the affinity of the solvent with that of semi-polar compounds. ^38^^,^^40^^,^^45^^,^^46^ Most of these studies yielded extracts with strong antioxidants (IC _50_ < 50 μg/mL). ^40^^,^^45^^,^^46^^,^^49^ Lipophilization using HCl or NaOH had been proven to yield higher amount of bioactive compounds and, as a consequence, increased the antioxidant properties. The antioxidant activity could also be improved by fermentation process using proper bacteria. ^47^^,^^50^ Nonetheless, extraction using more sophisticated solvent, such as supercritical CO _2_, did not contribute to higher anti-oxidant activity (IC _50_ > 250 μg/mL). ^48^

Since coffee by-products contain nutrients and antioxidants, they might be used as food additives or animal food sources. Simple ensiling and sun-drying were sufficient to produce fodder with high protein and fiber possessing antioxidant capacity as high as 2.5 μmol TE ^-1^ mL. ^37^ Dietary fiber had been isolated from various coffee by-products of both robusta and arabica variant containing bioactive compounds and high antioxidant activity. ^53^ Coffee by-products could also be used as food odorants and dye. ^6^^,^^44^^,^^51^ Beverage powder made of arabica cascara was proven to contain high amount of nutrients and antioxidant activity. ^43^ The use of coffee by-product in topical formulation, such as lotion, had been reported as well. ^46^

Phenolics, flavonoids, and tannins are among the common compounds found in aqueous extracts of coffee husks, coffee pulp, and coffee, silverskin, ^22^^,^^38^ which are identical to coffee bean. ^70^^,^^71^ Caffeine, gallic acid, and proanthocyanidins were also contained in the by-products, ^48^^,^^52^ contributing to the antioxidant efficacies. ^72^ Tannins could prevent the oxidative stress, oxidative damage, and UVB-induced matrix metalloproteinase-1. ^73^ Coffee cascara was reported to possess 8 times higher anti-radical capacity compared to blueberry with anti-cancer and vitality booster properties. ^52^ Other health benefits of coffee by-products could be attributed to their rich fiber, magnesium, calcium, and vitamin C, and low fat content. ^24^ Additionally, coffee cascara was also known to contain pectin which can be used as food additive. ^45^ The aqueous extract of arabica coffee pulp could inhibit the production of IL-8 in gastric epithelial cells. ^45^ Antibacterial properties of coffee cascara, coffee silverskin, and coffee husk have been reported as well. ^6^^,^^22^^,^^49^

Bread made of coffee husk and coffee silverskin was reported free of gluten, carrying an antioxidant, α glucosidase inhibitor, which is potential to reduce chronic diseases, oxidative stress, cholesterol level, and post prandial blood glucose level. ^74^ Arabica coffee pulp yielded high antibacterial activity against nosocomial bacteria; *Staphylococcus Epidermidis* and *Pseudomonas Aeruginosa*. ^75^ Fermented coffee pulp had high phenolic compounds and with pH level and total acid level. ^76^ Phenolic compounds are useful in reducing inflammation-associated cholesterol via adipogenesis inhibition. ^77^ Anti-cholesterol properties of coffee by-product was reported to effectively decrease the cholesterol level by inhibiting the absorbance of colonic cholesterol. ^75^

## Conclusions

Coffee by-products relatively have high antioxidant activities, depending on the processing method and variant. Other than antioxidants, they are rich in fiber and nutrients making them as potential additives in multiple products. Despite their high antioxidant activity and polyphenol content, the utilization of coffee by-products in topical formulation is relatively scarce compared to that in foods or beverages. Furthermore, investigation using different assays and parameters making it difficult to compare the results from each research. Hence, we recommend using robust and uniform methods in determining the antioxidant activity of coffee by-products.

## Data availability

### Underlying data

All data underlying the results are available as part of the article and no additional source data are required.

### Reporting guidelines

Figshare: PRISMA checklist for ‘Coffee by-products as the source of antioxidants: a systematic review’. DOI: https://doi.org/10.6084/m9.figshare.18866456.

Data are available under the terms of the Creative Commons Attribution 4.0 International license (CC-BY 4.0).

## Conflict of interest

The authors declare that they have no conflict of interest.

## Ethics statement

Not required.
