# Supplementary material for: Coffee by-products as the source of antioxidants: a systematic review
Source: F1000Res. 2022 Feb 23;11:220. [Version 1] doi: 10.12688/f1000research.107811.1 (PMC9123331; doi:10.12688/f1000research.107811.1)
Supplement: Peer review history

# Peer review: Reviewer response for version 1

**DOI:** 10.5256/f1000research.119070.r136770

**Published:** 2022-05-20

**Authors:** Ghodratollah Panahi

**Affiliations:** 1 Department of Biochemistry, Faculty of Medicine, Tehran University of Medical Sciences, Tehran, Iran

In this research, the authors systematically reviewed the studies that evaluated the antioxidant activity of coffee by-products obtained from different coffee variants. They provide evidence that, depending on the processing method and variant, coffee by-products have high antioxidant activities. It is suggested that these compounds could be used as additives in foods, beverages, and cosmetics but their benefits in skincare products require further investigation.

 The article is well written and methodologically well done. Congratulations to the authors of this research, and I think the article is ready to be accepted and published.

Are the rationale for, and objectives of, the Systematic Review clearly stated?

Yes

Is the statistical analysis and its interpretation appropriate?

Yes

Are sufficient details of the methods and analysis provided to allow replication by others?

Yes

Are the conclusions drawn adequately supported by the results presented in the review?

Yes

Reviewer Expertise:

Diabetes, endocrine disorder, miRNA, molecular Biology

I confirm that I have read this submission and believe that I have an appropriate level of expertise to confirm that it is of an acceptable scientific standard.

# Peer review: Reviewer response for version 1

**DOI:** 10.5256/f1000research.119070.r128542

**Published:** 2022-04-11

**Authors:** Emil Salim

**Affiliations:** 1 Faculty of Pharmacy, Universitas Sumatera Utara, North Sumatera, Indonesia

Coffee depulping processes generate solid waste that threatens the environment. Many researchers reported that coffee by-products have the potential as antioxidant sources. This systematic review aimed to assess the antioxidant activity of coffee by-products derived from various coffee varieties (arabica and robusta) and processing techniques. As of May 29, 2021, this study was carried out for papers published within 2011-2021 using Embase, Medline, BMJ, Web of Science, Science Direct, Cochrane, and PubMed databases.

 PRISMA guidelines were used to collect information on the type of specimen, processing procedures, and antioxidant activity. Aqueous extraction was the most common method to obtain the antioxidant from various coffee by-products, followed by methanolic and ethanolic extraction. The antioxidant properties varied depending on coffee by-products (cascara, pulp, husk, silverskin, and parchment) and processing method. Coffee by-products contain antioxidant compounds that could be employed as food, beverage, and cosmetic additives.

 This systematic review is critical for identifying, evaluating, and summarising the antioxidant activity of coffee by-products obtained from different coffee varieties (arabica and robusta) and processing procedures. The review’s rationale, objective, and method were clearly stated, and the selected reports support the conclusion.

 However, to improve the quality of this review, I suggest some corrections:

1. **“Results: **Our data suggested that aqueous extract was found to be the most common processing method used to obtain the antioxidant from various coffee by-products, followed by methanol and ethanol extract.” I suggest that aqueous extract…..methanol and ethanol extract are replaced by aqueous extraction…..methanol and ethanol extraction.
2. “Many studies have been conducted in coffee.” This sentence is unclear; please add more information about the studies.
3. "Phenolics, flavonoids, and tannins are among the common compounds found in aqueous extracts of coffee husks, coffee pulp, and coffee, silverskin, ^22,38^ which are identical to coffee bean." It should be 'coffee silverskin' (without a comma).
4. *Staphylococcus Epidermidis* and *Pseudomonas Aeruginosa *should be written as *Staphylococcus epidermidis* and *Pseudomonas aeruginosa*.

Are the rationale for, and objectives of, the Systematic Review clearly stated?

Yes

Is the statistical analysis and its interpretation appropriate?

Not applicable

Are sufficient details of the methods and analysis provided to allow replication by others?

Yes

Are the conclusions drawn adequately supported by the results presented in the review?

Yes

Reviewer Expertise:

Natural product, innate immunity

I confirm that I have read this submission and believe that I have an appropriate level of expertise to confirm that it is of an acceptable scientific standard.
